# Rational Design of Recombinant Papain-Like Cysteine Protease: Optimal Domain Structure and Expression Conditions for Wheat-Derived Enzyme Triticain-α

**DOI:** 10.3390/ijms18071395

**Published:** 2017-06-29

**Authors:** Neonila V. Gorokhovets, Vladimir A. Makarov, Anastasiia I. Petushkova, Olga S. Prokopets, Mikhail A. Rubtsov, Lyudmila V. Savvateeva, Evgeni Yu. Zernii, Andrey A. Zamyatnin

**Affiliations:** 1 Institute of Molecular Medicine, Sechenov First Moscow State Medical University, Trubetskaya str., 8, bld. 2, Moscow 119991, Russia; gorokhovets@gmail.com (N.V.G.); known.sir@yandex.ru (V.A.M.); ludmilaslv@yandex.ru (L.V.S.); 2 Lomonosov Moscow State University, Faculty of Biology, Moscow 119991, Russia; asyapeti@gmail.com (A.I.P.); ma_rubtsov@mail.ru (M.A.R.); 3 International Associated Laboratory (LIA) 1066 French-Russian Joint Cancer Research Laboratory, Villejuif 94805, France; 4 Institut für Pharmakologie and Klinische Pharmazie, Philipps-Universität Marburg, Marburg D-35043, Germany; olga.prokopets@pharmazie.uni-marburg.de; 5 Department of Biological Chemistry, Sechenov First Moscow State Medical University, Trubetskaya str., 8, bld. 2, Moscow 119991, Russia; 6 Belozersky Institute of Physico-Chemical Biology, Lomonosov Moscow State University, Moscow 119992, Russia; zerni@mail.ru

**Keywords:** recombinant protein, papain-like cysteine protease, protein folding, proteolytic cleavage, autocatalytic activation, celiac disease

## Abstract

Triticain-α is a papain-like cysteine protease from wheat (*Triticum aestivum* L.) that possesses activity towards toxic gluten-derived peptides, and was thus proposed as a novel therapeutic tool for celiac disease. We report an original approach employing rational design of domain architecture of Triticain-α and selection of the appropriate expression system for development of cheap and efficient protocol yielding active recombinant enzyme. The segregated catalytic domain of Triticain-α did not adopt native structure in bacteria, neither being expressed as a single protein nor upon conjugation or co-expression with extrinsic chaperones. Meanwhile, its attachment to prodomain of the enzyme resulted in generation of insoluble (inclusion bodies) product that can be transformed into active protease upon refolding in vitro. The estimated yield of the product was affected by affinity six-histidine tag required for its single-step purification with the preferable N-terminal position of the tag. Expression of the two-domain Triticain-α construct in yeast (*Pichia pastoris*) strain GS115 and bacterial (*Escherichia coli*) strain Rosetta gami B (DE3) led to the accumulation of a soluble protein, which underwent autocatalytic maturation during expression (in yeast)/purification (in bacteria) procedures and exhibited pronounced protease activity. Furthermore, expression and solubility of such construct in Rosetta gami B (DE3) cells was improved by reducing the temperature of the bacterial growth yielding more active enzyme than yeast counterpart presumably due to facilitated formation of a characteristic disulfide bond critical for maintaining the catalytic site. We suggest that these findings are helpful for obtaining active Triticain-α preparations for scientific or medical applications, and can be employed for the design and production of beneficial recombinant products based on other papain-like cysteine proteases.

## 1. Introduction

Recombinant proteases of exogenous origins are broadly used for therapeutic applications [1]. In particular, they were suggested for treatment of celiac disease (CD, also referred to as celiac sprue) as a promising alternative to strict gluten-free diet [2]. CD is a complex autoimmune disease characterized by an aberrant inflammatory response of the small intestine to dietary gluten from wheat and related proteins from rice and barley in genetically susceptible individuals [3]. It is common in South and North America, West and East Europe, North Africa, Southwest Asia, Siberia and Australia affecting approximately 1% of the population, whereas its prevalence is lower in the Far East [4,5]. Gluten is a heterogeneous mixture of insoluble storage proteins, gliadins, which contain proline-rich and glutamine-rich repetitive sequences and exhibit resistance to complete proteolysis by human digestive enzymes [6]. In CD patients, gluten-derived Pro/Gln-rich oligopeptides are accumulated in the lumen of the small intestine and induce HLA-DQ2-restricted or HLA-DQ8-restricted T-cell responses, thereby triggering inflammatory processes [7].

Various proteases (glutenases) capable of inactivating immunogenic gluten peptides in the human gastrointestinal tract can be used to treat CD [8]. For instance, a mixture of barley cysteine endoprotease EP-B2 and *Sphingomonas capsulata* prolylendopeptidase (PEP), designated in clinical trials as ALV003, is currently being developed for oral therapy of the disease [9]. Recently, wheat (*Triticum aestivum* L.) cysteine protease Triticain-α was proposed as a new therapeutic agent to treat CD. Original study by Kiyosaki and co-authors suggested that Triticain-α participates in seed maturation by digesting storage proteins during the germination [10]. Lately the enzyme was shown to possess glutenase activity in vitro at acidic (or close to neutral) pH levels at human body temperature. Remarkably, Triticain-α cleavage sites were found in the majority of the previously identified gluten-derived toxic peptides, including the major 33-mer α-gliadin-derived peptide generating inflammatory responses to gluten in CD patients [11]. Taken together, these data point to the great potential of Triticain-α as a basic compound for the development of pharmaceuticals effective in CD treatment.

As such, there is a demand for structural and functional evaluations of Triticain-α, as well as its pre-clinical and clinical trialing, which requires sufficient amounts of the enzyme. To date, several protocols for the production of recombinant papain-like cysteine proteases utilizing bacterial, yeast, baculovirus or mammalian cell culture expression systems have been described [12]. Baculovirus or mammalian cells possess benefits of eukaryotic expression systems, but their applications are still expensive in comparison to the production of proteins using yeast or bacteria. The advantages of the yeast expression systems are eukaryotic protein folding and posttranslational modification machinery, which, for instance, may allow for the correct processing and glycosylation. The additional advantage of the *Pichia*-based expression systems is the very low level of secretion of endogenous proteins into the medium that, in the case of expressing secretory heterologous products, simplifies their purification procedure. However, expression of recombinant proteins in *Escherichia coli* is usually the most preferred due to the high degree of elaboration, rapid growth of biomass and relatively low cost. Meanwhile, in *E. coli*, most recombinant proteins are produced in insoluble form, and thereby require extra efforts to accomplish recombinant protein refolding, which is difficult to scale and often reduces the target product yield. In this respect, papain-like proteases are no exception since most attempts to perform their heterologous expression in *E. coli* resulted in production of the recombinant protein in insoluble form [12]. Furthermore, their in vitro refolding is commonly associated with simultaneous maturation of an active enzyme, which undergoes autocatalytic degradation. These factors concertedly complicate validation of protocols for papain-like proteases production using *E. coli*-based expression systems.

**Figure 1:**
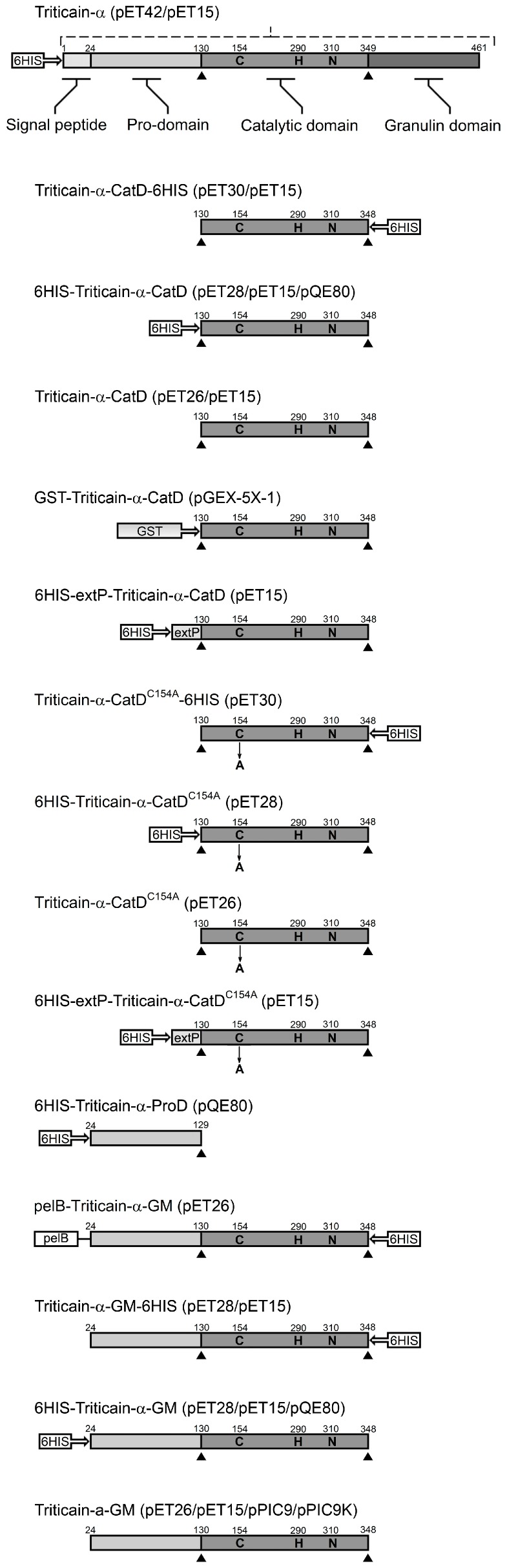
Schematic representation of Triticain-α constructs. Vectors employed for expression of the corresponding genetic constructs in *E. coli* and *P. pastoris* are indicated in round brackets. Predicted cysteine protease catalytic residues Cys-154 (or its substitutive Ala), His-290 and Asn-310 are shown as C(A), H and N. 6HIS, N-terminal or C-terminal six-histidine tag; CatD, Triticain-α catalytic domain; extP, 16-mer folding peptide (NYEEVIKKYRGEENF) from *Plasmodium falciparum* cysteine protease falcipain-2; GST, glutathione *S*-transferase; pelB, N-terminal bacterial periplasmic signal sequence; ProD, Triticain-α pro-domain; Triticain-α-GM, Triticain-α lacking signal peptide sequence and region for granulin domain.

The solution of these problems is based not only on adequate selection of the expression system for each protease, but also on optimization of its structure to increase solubility of the product and prevent its unwanted degradation. Indeed, for most cysteine protease genes, the translational products consist of catalytic domain supplied by autoinhibitory prodomain preventing untimely activation of the enzyme. Plant-encoded papain-like enzymes additionally contain extensions of these domains, such as N-terminal signal peptide, C-terminal Pro-rich domain, C-terminal granulin-like domain, or *C*-terminal retrieval signal for localization to the endoplasmic reticulum (K/HDEL) [13]. Consistently, full primary structure of Triticain-α along with autoinhibitory and protease domains contains *N*-terminal signal peptide and C-terminal granulin-like domain (Figure 1) [10]. A number of experimental evidences demonstrated that these additional structural elements are not necessarily required for the proper maturation of an active enzyme in vitro [11,14]. Indeed, the maturation of Triticain-α can occur because of autocatalytic activation of the purified enzyme initially lacking N-terminal signal peptide and granulin-like domain (“Triticain-α-GM”, Figure 1). Although Triticain-α-GM is efficiently expressed in *E. coli* (up to 30–50 mg/L of culture), it is accumulated in insoluble inclusion bodies [11]. Thus, the obtaining of the active enzyme mandatory included combination of Ni-NTA chromatography of the denaturated protein and its subsequent refolding, which, as noted above, is a time-consuming process associated with autocatalytic activation of the enzyme, and can be poorly controlled. Considering all these observations, in the present study, we have focused on the rational design of the optimal domain architecture of Triticain-α and selection of expression system to develop potentially scalable protocol for cheap and efficient production of active recombinant enzyme.

## 2. Results and Discussion

### 2.1. Expression of Full-Length Triticain-α in E. coli

Keeping in mind the outstanding time and cost efficiency of protein production using bacterial expression systems, our primary efforts were aimed at optimizing domain structure of Triticain-α to increase solubility of the respective product in *E. coli*. In the first step, we assessed bacterial expression of full-length Triticain-α (Figure 1), assuming that the extra elements found in primary structure plant-encoded proteases may facilitate its folding and increase its solubility. For this purpose, Triticain-α gene was subcloned into plasmid pET42b(+) providing pronounced expression of a recombinant gene under the control of the T7 RNA polymerase promoter and *lac* operator. The gene was inserted into the vector in a way to obtain N-terminally 6HIS-tagged product that can be easily purified. *E. coli* strain BL21 (DE3) was chosen as the primary host since it has the advantage of being deficient in both *lon* and *ompT* proteases and is compatible with the T7 *lac*O promoter system. Analytical expression revealed accumulation of protein with molecular weight of approximately 60 kDa (corresponding to 6HIS-tagged full-length Triticain-α) with product yield of 23 mg/L of culture after 4 h of cultivation (Table 1). Further fractionation analysis indicated that almost all protein transferred into inclusion bodies. Furthermore, all our attempts to purify the protein using Ni-NTA column after extraction from insoluble fraction failed. Thus, in contrast to Triticain-α-GM, the full-length enzyme is likely incapable of binding to histidine-affinity matrices and therefore further studies of this form were abandoned.

### 2.2. Expression of Catalytic Domain of Triticain-α in E. coli

**Figure 2:**
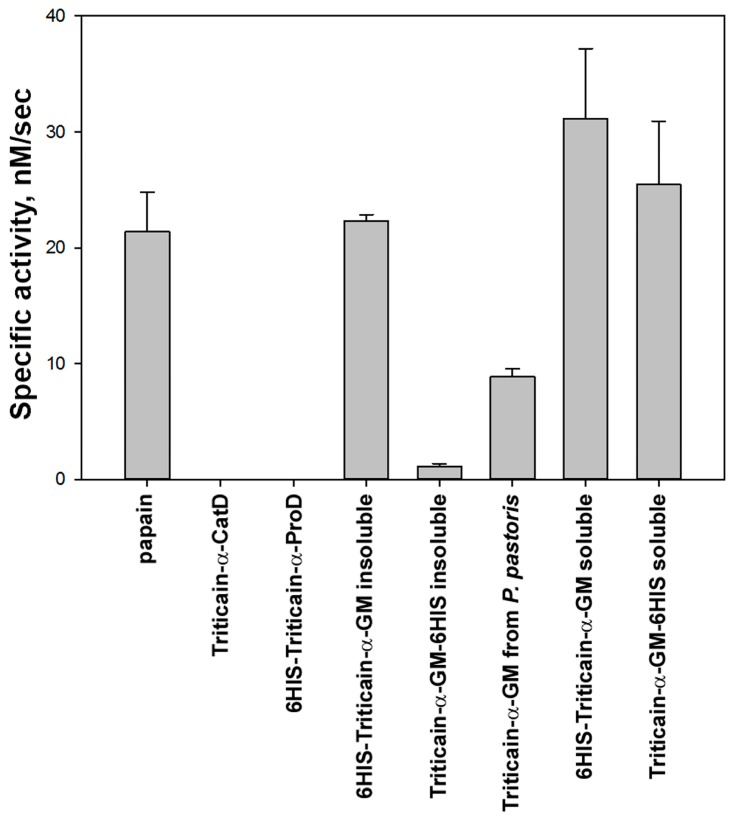
Protease activity of Triticain-α derivatives. The enzymatic reaction rates were determined from the initial slope of the progress curves registered in fluorescent protease activity assay utilizing peptide substrate acetyl-(Pro-Leu-Val-Gln)-7-amino-4-methylcoumarin (Ac-PLVQ-AMC). Activity of papain was used as a control. Error bars denote the standard error of triplicate measurements.

It is well established that low molecular weight proteins are preferred by *E. coli* expression systems [15]. Therefore, to reduce the size of the expression product in the next set of experiments, we created pET-based genetic constructs encoding single catalytic domain of Triticain-α (Triticain-α-CatD, *M*r = 23.57 kDa, Figure 1). One of the basic requirements for the Triticain-α obtaining technique was its single-step purification from the cellular lysates, which needs an affinity tag. Meanwhile, the presence and localization of 6HIS-tag is known to affect expression level and solubility of recombinant proteins in *E. coli* [16]. Thus, in our constructs, the tag was positioned either on the N-terminal or the C-terminal end of Triticain-α-CatD, or was absent. In order to segregate alterations in expression of Triticain-α-CatD from its potential autocatalytic proteolysis in vivo, the same forms containing inactivating mutation of catalytic cysteine (C154A) were additionally created. Finally, we obtained three more constructs, namely Triticain-α-CatD fused to glutathione S-transferase (GST) and Triticain-α-CatD/Triticain-α-CatD^C154A^ fused to 16-mer folding domain (NYEEVIKKYRGEENF, “extP”) of cysteine protease falcipain-2 from *Plasmodium falciparum* [17]. GST is known to increase the solubility of different protein fragments in bacteria, while extP produced such effect on adjacent catalytic domain of falcipain-2 [17,18]. Analysis of expression of the obtained constructs in *E. coli* strain BL21 (DE3) revealed efficient accumulation of all forms of Triticain-α-CatD regardless of the presence of C154 mutation. Interestingly, expression of N-terminally tagged Triticain-α-CatD exceeded C-terminally tagged protein fivefold and was 10-fold more pronounced compared to the domain lacking 6HIS-tag (Table 1). These data indicate that the sequence of N-terminally tagged Triticain-α-CatD is optimal for expression in bacteria. Similar high-yield production was observed in the case of GST-tagged and extP-conjugated proteins apart from 6HIS-extP-Triticain-α-CatD (Table 1). Even so, all variants of Triticain-α-CatD entered inclusion bodies and remained in insoluble fraction during extraction by urea-free buffer. Furthermore, none of these forms could digest model fluorescent substrate acetyl-(Pro-Leu-Val-Gln)-7-amino-4-methylcoumarin (Ac-PLVQ-AMC) in fluorescent protease activity assay after affinity purification under denaturating conditions and refolding in vitro (Figure 2 and data not shown). Thus, employment of the constructs encoding single catalytic domain of Triticain-α can significantly increase expression of the recombinant product, but its structure is not sufficient for the maturation of the active enzyme.

### 2.3. Co-Expression of Catalytic Domain of Triticain-α in E. coli with Folding Chaperones

We further assumed that the correct tertiary structure of Triticain-α catalytic domain providing full activity can be adopted in the presence of folding chaperones, among which one can firstly consider its natural prodomain. To assess whether the latter being expressed as separate protein can exhibit such chaperone-like activity, we obtained the respective construct (Triticain-α-proD, *M*r = 12.71 kDa) and investigated its ability to facilitate Triticain-α-CatD folding in vivo. As expected, 6HIS-tagged Triticain-α-ProD was at least partially produced as soluble protein. However, its co-expression with Triticain-α-CatD or the respective inactivating mutant did not increase the solubility regardless of the presence and position of 6HIS-tag (Table 2). Consistently, the soluble fractions obtained after co-expression of Triticain-α-proD with wild type Triticain-α-CatD displayed no activity in Ac-PLVQ-AMC fluorescence assay (data not shown). We further attempted to facilitate Triticain-α-CatD folding by its co-expression with human heat shock protein 70 (HSP70A1B), a well-recognized intracellular chaperone [19]. Although HSP70 itself was produced in *E. coli* BL21 (DE3) cells as a cytoplasmic protein, it induced no transfer of Triticain-α-CatD into the soluble fraction (Table 2).

All these data taken together indicate that the correct folding of active catalytic domain of Triticain-α cannot be facilitated by extrinsic chaperones and probably require expression with other domain(s) of the enzyme as single polypeptide chain. Among these domains, the granulin-like domain was shown to be dispensable for activity of cysteine proteases and, furthermore, renders the enzymes prone to precipitation [11,20,21,22]. Consistently, the members of only two out of the nine subfamilies of plant-encoded papain-like cysteine proteases (subfamily 1 and 4) harbor such C-terminal extension [13]. By contrast, their prodomain was demonstrated to be necessary for maturation of the catalytically active enzyme [11,21,23]. Moreover, for some cysteine proteases the prodomain was reported to function as an intramolecular chaperone promoting proper folding of the mature enzyme [24]. These observations forced us to return to using two-domain Triticain-α-GM as a template for the further improvement of expression, solubility and activity of the recombinant product.

### 2.4. Expression of Two-Domain Triticain-α Constructs in E. coli

As mentioned above, the basic sequence in Triticain-α-GM construct is deprived of *N*-terminal signal peptide and granulin-like domain, i.e. consists of prodomain and catalytic domain of the enzyme (Triticain-α-GM, *M*r = 36.27 kDa, Figure 1). Since the presence and position of 6HIS-tag affected expression of Triticain-α-CatD (see above), we rechecked if the same is valid for two-domain Triticain-α. To this end, genetic constructs encoding *N*-terminally tagged, *C*-terminally tagged, and untagged Triticain-α-GM in pET vectors were created (Figure 1). It is known that expression in bacteria and purification of the recombinant proteins may be improved by inducing their secretion into the extracellular medium [25]. Therefore, we additionally produced C-terminally tagged Triticain-α-GM containing bacterial periplasmic signal sequence (pelB) on its N-terminus (Figure 1).

All variants of Triticain-α-GM, including pelB-Triticain-α-GM, were expressed in *E. coli* BL21 (DE3) cells exclusively in inclusion bodies (Table 1). Reduction of the cell growth temperature that is commonly recommended to improve the solubility of recombinant proteins in bacteria produced no effect in this case (data not shown). Notably, Triticain-α-GM variants exhibited different production levels with N-terminally tagged protein yield being 2–3-fold higher than for any of the other constructs. Refolding of the insoluble fractions in vitro resulted in maturation of an active protease regardless of the presence or position of 6HIS-tag. However, the specific proteolytic activity of the enzyme with C-terminal tag and the one lacking the tag was extremely low suggesting that only a minor fraction of the protein molecules recovered their tertiary structure in these cases. By contrast, 6HIS-Triticain-α-GM exhibited pronounced activity comparable to that of papain taken as a reference cysteine protease (Figure 2). In sum, these data indicate that the two-domain structure of the enzyme containing N-terminal 6HIS-tag is necessary and sufficient for protease maturation.

### 2.5. Expression of Triticain-α Constructs in Yeast

**Figure 3:**
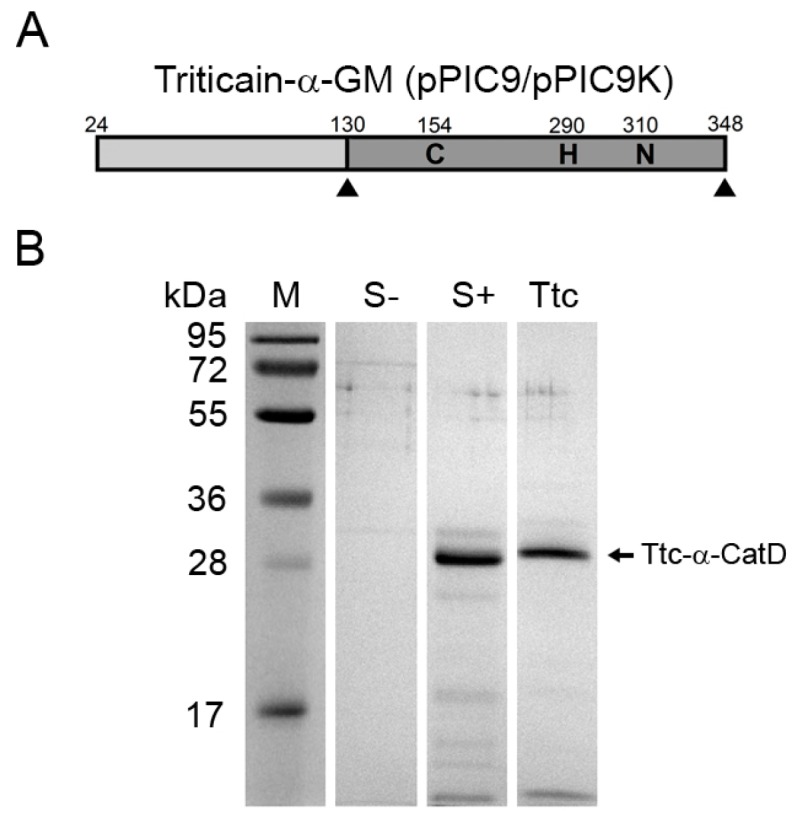
Expression and purification of soluble Triticain-α using yeast expression system: (**A**) schematic representation of domain structure of Triticain-α construct employed for the expression in *P. pastoris*; and (**B**) sodium dodecyl sulphate polyacrylamide gel electrophoresis (SDS-PAGE) of *P. pastoris* cell growth medium after the cultivation of Triticain-α transformants with (“S+”) or without (“S−“) methanol induction; “Ttc” – purified Triticain-α-GM. The positions of molecular weight markers are indicated (“M”).

It could be suggested that the isolation of correctly folded 6HIS-Triticain-α-GM into inclusion bodies during cell growth is associated with specific effects of the protein on the employed bacterial strain and can be suppressed by altering expression conditions or the host organism. With that in mind, we firstly changed expression system to yeast *P*. *pastoris*. GS115 strain was transformed by pPIC9 vector bearing DNA fragment encoding untagged Triticain-α-GM (Figure 3A) in such a way as to obtain Mut^+^ and Mut^S^ yeast phenotypes characterized by fast and slow rates of cell growth on methanol-containing medium. Analytical expression of the resulting yeast clones revealed more potent accumulation of recombinant protein in the growth medium in the case of Mut^S^ phenotype (Table 1). Notably, the molecular weight of the major product in both cases was about 31 kDa, which corresponds to the mature form of the enzyme (Figure 3B). Thus, Triticain-α-GM was capable of autocatalytic maturation, which means that it underwent correct folding in yeast. Consistently, only minor fraction of full-length protein with *M*r ~43 kDa was detected upon concentrating of the growth medium samples (data not shown). Although the purity and total yield of the recombinant enzyme were high (up to 170 mg/L, Figure 3B), its specific activity in fluorescent protease activity assay was quite low, barely reaching a half of that for papain or 6HIS-Triticain-α-GM refolded in vitro (Figure 2). The double transformation of GS115 strain by pPIC9 and pPIC9K vectors increased estimated yields to approximately 170 and 300 mg/L for Mut^+^ and Mut^S^ phenotypes, respectively. However, such modification of the procedure merely affected protease activity of the product. Thus, the moderate activity of yeast-derived Triticain-α-GM along with low cost-efficiency of its production in yeast may limit feasibility of this expression system for the obtaining of the recombinant enzyme.

### 2.6. Selection of Bacterial Strain for Expression of Triticain-α Constructs in Soluble Form

**Figure 4:**
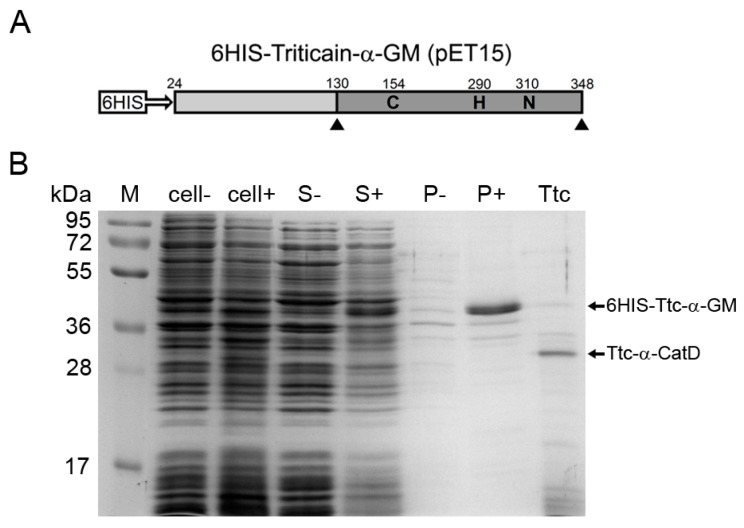
Expression and purification of soluble Triticain-α using bacterial expression system: (**A**) Schematic representation of domain structure of Triticain-α construct employed for the expression in *E. coli* strain Rosetta gami B (DE3); and (**B**) sodium dodecyl sulphate polyacrylamide gel electrophoresis (SDS-PAGE) of cellular lysates (“cell”), extracts (“S”) or insoluble fractions (“P”) obtained from Triticain-α transformants with (+) or without (−) IPTG induction; Ttc – purified Triticain-α-GM (the positions of zymogene and mature enzyme are indicated). “M” denotes molecular weight markers.

Given the aforementioned findings, we further attempted to optimize bacterial expression of Triticain-α-GM in order to improve solubility of the product. For this purpose, we used two *E. coli* strains alternative to BL21 (DE3), namely JM109 and Rosetta gami B (DE3). JM109 cells were previously shown to efficiently express recombinant proteins in soluble form [19,26]. The benefits of Rosetta gami B (DE3) are that it provides six codons for rare tRNAs and carries the *gor* and *trx* mutations, which facilitate the formation of intramolecular disulfide bonds in proteins thereby improving their folding and solubility [27]. The latter feature of Rosetta gami B (DE3) could be important for the production of papain-like proteases since they contain a characteristic disulfide bond (C159–C201 in papain [28,29] that corresponds to C151–C186 in Triticain-α), which is critical for maintaining their catalytic site [30]. Consistently, previous studies demonstrated feasibility of this *E. coli* strain for expression of active cysteine proteases [27]. The genetic constructs encoding high-yielding N-terminally tagged Triticain-α-GM (Figure 4A) and Triticain-α-CatD were introduced in pQE80L and pET15b vectors to be expressed in JM109 and Rosetta gami B (DE3), respectively. Fractionation analysis revealed that 6HIS-Triticain-α-GM was partially produced in Rosetta gami B (DE3) as a soluble protein. With that in mind, we further examined Rosetta gami B (DE3) expression patterns for full-length Triticain-α as well as Triticain-α-GM and Triticain-α-CatD bearing 6HIS-tag in different positions (Table 1). In addition, we monitored folding of 6HIS-Triticain-α-CatD upon its co-expression with 6HIS-Triticain-α-proD (Table 2). Among the catalytic domain-containing forms of the enzyme, only Triticain-α-GM accumulated in the soluble fraction independently of the tag location (Figure 4B). Furthermore, in contrast to BL21 (DE3) strain, the reduction of cell growth temperature from 37 to 18 °C in the case of Rosetta gami B (DE3) cells further enhanced solubility of Triticain-α-GM variants. It should be noted, that the estimated yield of these proteins was lower compared to refolded counterparts (Table 1), but this disadvantage was compensated by their increased enzymatic activity (Figure 2). Indeed, Rosetta gami B (DE3)-derived Triticain-α-GM constructs were easily purified using non-denaturating Ni-NTA chromatography, yielding predominantly 31 kDa product with an admixture of proteolytic fragments, formation of which is indicative of autocatalytic maturation of the enzyme (Figure 4B). Both forms of Triticain-α-GM potently catalyzed Ac-PLVQ-AMC digestion and the soluble 6HIS-Triticain-α-GM was the most effective in this respect since its specific activity by a half-exceeded activity of its refolded analog or papain (Figure 2). Furthermore, the enzyme production from plasmid to protein in this case was less time-consuming compared to refolding in vitro, and therefore its catalytic activity can be more easily controlled during the obtaining procedure. Overall, the expression of N-terminally tagged two-domain Triticain-α in bacterial strain Rosetta gami B (DE3) followed by single step affinity purification of the recombinant product was considered as an optimal method for the obtaining of catalytically active enzyme.

As exemplified by Triticain-α, optimization of domain structure of a cysteine protease along with rational selection of appropriate expression system can significantly improve procedure and cost-effectiveness of an active recombinant enzyme production. In the case of Triticain-α, it was found that: (i) minimal structure of the enzyme required for its folding in vivo contains its prodomain covalently attached to the catalytic domain (Triticain-α-GM); (ii) conjugation or co-expression of the catalytic domain of Triticain-α with extrinsic chaperones (GST, extP, and HSP70) does not improve folding of the enzyme; (iii) N-terminal position of the affinity six-histidine tag in Triticain-α-GM is more preferable than its C-terminal position; (iv) expression of the Triticain-α-GM as soluble protein results in the generation of more catalytically active protease than in the case of its refolding after expression in inclusion bodies; (v) soluble Triticain-α-GM is produced both in bacterial (*E. coli* strain Rosetta gami B (DE3)) and yeast (*P*. *pastoris* strain GS115) expression systems (Figure 3 and Figure 4); and (vi) expression and solubility of Triticain-α-GM in Rosetta gami B (DE3) can be improved by reducing temperature of the bacterial growth and yields more active enzyme presumably due to facilitated formation of disulfide bonds in the cytoplasm of such cells. We suggest that these findings are helpful to obtain Triticain-α preparations for scientific purposes or medical applications such as the production of pharmaceuticals for the treatment of CD. In addition, they can be employed to design and produce beneficial recombinant products based on the other papain-like cysteine proteases.

## 3. Materials and Methods

### 3.1. Materials

Papain, BCA kit and other reagents were purchased from Sigma-Aldrich (St. Louis, MO, USA) except where noted. Tryptone, peptone, yeast extract and agar were from Becton, Dickinson and Company (Franklin Lakes, NJ, USA), ethanol was from Merck Millipore (Billerica, MA, USA), methanol was from Honeywell Burdick & Jackson (Bucharest, Romania), acetonitrile was from PanReac (Barcelona, Spain). Advantage 2 Polymerase Mix was purchased from Takara Bio Inc. (Shiga, Japan). Other enzymes used for cloning procedures were from Thermo Fisher Scientific Inc. (Waltham, MA, USA). Sephacryl S-200HR and nickel-nitriloacetic acid (Ni-NTA) sepharose was purchased from GE Healthcare Life Sciences (Pittsburgh, PA, USA). Peptide acetyl-Pro-Leu-Val-Gln conjugated with 7-amino-4-methylcoumarin (Ac-PLVQ-AMC) was ordered from Pepmic Co., Ltd. (Suzhou, China).

### 3.2. Genetic Constructs for Expression in E. coli

Expression vectors encoding Triticain-α variants were based on commercially available plasmids pET15b, pET26b(+), pET28a(+), pET30a(+), pET42a(+) (Merck Millipore, Billerica, MA, USA), pQE80L (Qiagen, Hilden, Germany), and pGEX-5X-1 (GE Healthcare Life Sciences, Pittsburgh, PA). Expression vector pQE80-6HIS-HSP70A1B as well as plasmids pET42-6HIS-Triticain-α, pQE80-6HIS-Triticain-α-GM, and pQE80-6HIS-Triticain-α-GM^C154A^ were obtained previously [11,31]. Molecular cloning was performed according to the standard protocols [32]. The aforementioned Triticain-α-encoding plasmids were used as templates for PCR amplification of *Triticain-α* fragments with or without C154A substitution. The oligonucleotides used in cloning procedures are summarized in Table S1.

To obtain pET26-Triticain-α-GM, pET28-6HIS-Triticain-α-GM, pET28-Triticain-α-GM-6HIS and pET26-pelB-Triticain-α-GM-6HIS, the corresponding PCR products were subcloned into pET26b(+) or pET28a(+) using *Nde*I-*Hind*III, or *Nco*I-*Xho*I, respectively. pQE80-6HIS-Triticain-α-ProD was produced via amplification of DNA fragment encoding Triticain-α prodomain and its subcloning into pQE80L using *Bam*HI and *Hind*III. Specific PCR product containing the sequence of *Triticain-α* catalytic domain was subcloned in pET-28а(+) and pET-26b(+) using *Nde*I-*Hind*III to produce pET28-6HIS-Triticain-α-CatD and pET26-Triticain-α-CatD. Alternatively, catalytic domain-containing DNA fragments were introduced into pQE80L (using *Bam*HI-*Hind*III), pET30a(+) (using *Nde*I-*Xho*I) and pGEX-5X-1 (using *Bam*HI-*Xho*I) yielding pQE80-6HIS-Triticain-α-CatD, pET30-Triticain-α-CatD-6HIS and pGEX-GST-Triticain-α-CatD, respectively. The expression vectors pET28-6HIS-Triticain-α-CatD^C154A^, pET30-Triticain-α-CatD^C154A^-6HIS and pET26-Triticain-α-CatD^C154A^ encoding Triticain-α derivatives with C154A mutation were generated similarly to the expression vectors for intact forms, except that the PCR products were obtained using the mutated template (see above). pET15-6HIS-Triticain-α was created by the ligation of *Xba*I-*Bam*HI DNA fragment excised from pET42-6HIS-Triticain-α into pET15b digested with the same restriction enzymes. Similarly, pET15-6HIS-Triticain-α-CatD was constructed by the ligation of *Xba*I-*Xho*I DNA fragment excised from pET28-6HIS-Triticain-α-CatD into *Xba*I-*Xho*I-digested pET15b. To obtain pET15-Triticain-α-GM and pET15-Triticain-α-CatD, appropriate *Xba*I-*Xho*I DNA fragments excised from pET26-Triticain-α-GM and pET26-Triticain-α-CatD were subcloned into *Xba*I-*Xho*I-digested pET15b. To produce pET15-6HIS-Triticain-α-GM the corresponding PCR product was subcloned into pET15b using *Nde*I and *Xho*I. The expression vector pET15-Triticain-α-GM-6HIS was obtained by simultaneous ligation of two DNA fragments resulting from the *Nco*I-*Pst*I or *Pst*I-*Xho*I digestion of PCR product of *Triticain-α-GM* amplification into *Nco*I-*Xho*I-digested pET15b. Similarly, three-component ligation was employed to prepare pET15-Triticain-α-CatD-6HIS wherefore the corresponding PCR product was digested with *Nco*I-*Pst*I or *Pst*I-*Xho*I and the resulting DNA fragments were ligated into *Nco*I-*Xho*I-digested pET15b. To prepare pET15-6HIS-extP-Triticain-α-CatD and pET15-6HIS-extP-Triticain-α-CatD^C154A^ vectors, the specific oligonucleotides (Table S1) were annealed and extended by Klenow enzyme to give a double-stranded DNA fragment encoding extP (NYEEVIKKYRGEENF). The fragment was then subcloned into pET15-6HIS-Triticain-α-CatD or pET28-6HIS-Triticain-α-CatD^C154A^ using *Nde*I. Dual gene vector pET15[6HIS-Triticain-α-CatD+6HIS-Triticain-α-ProD] was produced by amplifying full Triticain-α-ProD-containing expression cassette of pQE80-6HIS-Triticain-α-ProD and subcloning the PCR product into pET15-6HIS-Triticain-α-CatD using *Bgl*II. Schematic representations of ORFs within all Triticain-α expression vectors are demonstrated in Figure 1.

### 3.3. Genetic Constructs for Expression in P. pastoris

*Triticain-α* variants were introduced in multicopy integration and secreted expression vectors pPIC9 and pPIC9K (Thermo Fisher Scientific). pPIC9-Triticain-α-GM was created by amplification of the *Triticain-α-GM*-containing DNA fragment using pET42-6HIS-Triticain-α as a template and subsequent subcloning of the PCR product into pPIC9 using *Eco*RI and *Not*I. To obtain pPIC9K-Triticain-α-GM, internal *Sal*I restriction site within *Triticain-α-GM* sequence was eliminated by site-directed mutagenesis using megaprimer method [33] in two rounds of PCR, where pET42-6HIS-Triticain-α was employed as a template. The final PCR product was subcloned into pPIC9K using *Eco*RI and *Not*I.

### 3.4. Expression and Co-Expression of Recombinant Proteins in E. coli

Transformed *E. coli* strains BL21 (DE3) or JM109 were cultivated in LB medium (10 g/L tryptone, 5 g/L yeast extract, 5 g/L NaCl), containing antibiotic (50 μg/mL ampicillin for pQE and pET15 transformants, or 30 μg/mL kanamycin for the rest of pET transformants, or both antibiotics for pET+pQE double transformants), at 37 °C with shaking at 250 rpm until the culture reached an optical density (OD_600_) of 0.6–0.8. The expression was induced by the addition of isopropyl thio-β-d-thiogalactopyranoside (IPTG) to a final concentration of 1 mM, and cells were incubated either at 37 °C for 3.5 h or at 28 °C for 15 h. For protein expression in *E. coli* strain Rosetta gami B (DE3), the transformed cells were cooled to 4 °C and incubated at 18 °C for 20 h after IPTG addition.

To analyze protein production and solubility, cells were harvested by centrifugation at 4000× *g*, 4 °C for 15 min. The cell pellets obtained from growth culture with OD_600_ of 3 were suspended in buffer A (50 mM Tris-HCl pH 7.8, 0.4 M NaCl, 1 mM EDTA, 1% Tween 20) followed by lysis using sonication. Supernatant (soluble fraction) was collected for the analysis by SDS-PAGE, whereas the pellet (insoluble fraction) was washed for three times using ice-cold 500 µL of buffer B (50 mM Tris-HCl pH 7.8, 0.4 M NaCl, 1 mM EDTA, 2 M urea) and resuspended in 300 µL of buffer A for further analysis with SDS-PAGE [34]. Quantitative analysis of protein content in the gels was performed using ChemiDoc MP Imaging System (BioRad, Hercules, CA, USA) according to the manufacturer’s recommendations. Specific productivity was calculated as a quantity of recombinant protein produced in growth culture with OD_600_ of 1 optical unit. Estimated yield indicates amount of recombinant protein produced in 1 L of growth culture.

### 3.5. Expression of Recombinant Proteins in P. pastoris

The GS115 (His^−^, Mut^+^) strain cells of *P*. *pastoris* were grown, transformed and selected following recommendations described in Pichia Expression Kit User Guide (Thermo Fisher Scientific Inc., Waltham, MA, USA). Briefly, *Bgl*II linearized pPIC9-Triticain-α-GM was used for the electroporation of *P*. *pastoris*. Mut^s^ or Mut^+^ transformants containing Triticain-α-GM inserts were selected on agar plate with minimal methanol medium. To obtain double transformants, Mut^s^ or Mut^+^ transformants were additionally electoporated with *Sal*I linearized pPIC9K-Triticain-α-GM. Further selection of obtained transformants was performed with geneticin (0.15 mg/mL) containing media.

To analyze protein expression, single *P*. *pastoris* colony was used to inoculate BMGY (buffered minimal glycerol-yeast, 1% (*w*/*v*) yeast extract, 2% (*w*/*v*) peptone, 1.34% (*w*/*v*) YNB, 4 × 10^−5^% (*w*/*v*) biotin, 1% (*v*/*v*) glycerol, 100 mM potassium phosphate buffer pH 6.0) medium. Cells were cultivated at 30 °C in an incubator shaker at 300 rpm until OD_600_ reaches 1.0 (for Mut^+^) or 5.0 (for Mut^s^). *AOX* promoter-controlled expression was induced by the replacement of BMGY with methanol-containing BMMY (buffered methanol-complex medium, 1% (*w*/*v*) yeast extract, 2% (*w*/*v*) peptone, 1.34% (*w*/*v*) YNB, 4 × 10−5% (*w*/*v*) biotin, 0.5% (*v*/*v*) methanol, 100 mM potassium phosphate buffer pH 6.0). Addition of methanol was reiterated each 24 h to the final concentration of 0.7% to maintain induction during cultivation time. Cell culture supernatants were recovered by centrifugation for 5 min at 4000× *g* (4 °C) and protein content was analyzed by SDS-PAGE [34]. Quantitative analysis of protein content in the gels was performed using ChemiDoc MP Imaging System (BioRad).

### 3.6. Purification of Recombinant Proteins

Soluble Triticain-α variants were purified from *E. coli* cellular extracts prepared by suspending biomass in PB (0.02 M sodium phosphate buffer, pH 8.0) containing 500 mM NaCl and 10 mM imidazole (Buffer C) followed by sonication (12 × 5 s) and centrifugation (10,000× *g*, 15 min). The extracts were applied onto a column with Ni-nitrilotriacetic acid (Ni-NTA) sepharose equilibrated with Buffer C and the bound protein was eluted with the same buffer, containing 300 mM imidazole. The resulting fraction was dialyzed against PB for 24 h.

For purification of the insoluble Triticain-α forms, the *E. coli* cells were washed five times by suspending in PB containing 130 mM NaCl, sonication (12 × 5 s) and centrifugation (10,000× *g*, 15 min) and the final pellet was dissolved in PB containing 300 mM NaCl, 10 mM imidazole and 8 M urea (Buffer D). The resulting fraction was sonicated, centrifuged (10,000× *g*, 30 min) and the supernatant was applied onto a column with Ni-NTA sepharose equilibrated with Buffer D. The bound protein was eluted with Buffer D containing 300 mM imidazole, diluted 5 times by PB, and refolded by repetitive dialysis against PB for 24 h. Alternatively, the refolding was performed during chromatography by washing sorbent with PB, containing 300 mM NaCl and 10 mM imidazole and eluting protein by the same buffer containing 250 mM imidazole. In all cases, the dialysates were concentrated in Amicon stirred cell supplied with a PM-10 membrane (MW cutoff 10 kDa, Merck Millipore, Billerica, MA USA) and the purified protein was quantified by the BCA kit, and lyophilized by freeze dryer. All purification procedures were performed at 4 °C and controlled by SDS-PAGE [34].

To obtain Triticain-α forms expressed in *P*. *pastoris*, the supernatant collected by centrifugation of yeast culture medium was dialyzed against PB for 24 h, the dialysate was concentrated and applied onto a column with Sephacryl S-200HR equilibrated with PB containing 130 mM NaCl at a flow rate of 30 mL/h. The 6 mL fractions were collected and analyzed for the presence of the target enzyme by SDS-PAGE and protease activity assay. The purified protein was concentrated, quantified and lyophilized as described above.

### 3.7. Fluorescent Protease Activity Assay

The activity of papain or Triticain-α variants was examined in the presence of the peptide substrate Ac-PLVQ-AMC (PLVQ sequence was selected based on analysis of Triticain-α-recognized sites in gluten proteins or collagen [11]) by monitoring fluorescence of the released AMC as a function of time. The assay was conducted at 25 °C in reaction mixture containing 20 nM enzyme, 50 µM Ac-PLVQ-AMC and 200 mM acetate buffer (pH 5.6), 100 mM NaCl, 15 mM 2-mercaptoethanol, 0.6 mM EDTA, 0.5% DMSO. The fluorescence was monitored at excitation wavelength of 360 nm and emission wavelength of 460 nm using GloMax-Multi Detection System (Promega, Madison, WI, USA). The reaction rates were determined from the initial slope of the progress curves by linear regression. The arbitrary fluorescence units were converted into the amount of hydrolyzed substrate by using a standard curve generated from the fluorescence measurements of the defined AMC concentrations. All enzymatic reactions were carried out in triplicate.

## 4. Conclusions

In this study we demonstrated that optimization of domain structure of a papain-like cysteine protease along with rational selection of appropriate expression system can significantly improve procedure and cost-effectiveness of an active recombinant enzyme production. Thus, in the case of Triticain-α , it was found that: (i) minimal structure of the enzyme required for its folding in vivo contains its prodomain covalently attached to the catalytic domain (Triticain-α-GM); (ii) conjugation or co-expression of the catalytic domain of Triticain-α with extrinsic chaperones (GST, extP, and HSP70) does not improve folding of the enzyme; (iii) N-terminal position of the affinity six-histidine tag in Triticain-α-GM is more preferable than its C-terminal position; (iv) expression of the Triticain-α-GM as soluble protein results in the generation of more catalytically active protease than in the case of its refolding after expression in inclusion bodies; (v) soluble Triticain-α-GM is produced both in bacterial (*E. coli* strain Rosetta gami B (DE3)) and yeast (*P. pastoris* strain GS115) expression systems (Figure 3 and Figure 4); and (vi) expression and solubility of Triticain-α-GM in Rosetta gami B (DE3) can be improved by reducing temperature of the bacterial growth and yields more active enzyme presumably due to facilitated formation of disulfide bonds in the cytoplasm of such cells. We suggest that these findings are helpful to obtain Triticain-α preparations for scientific purposes or medical applications such as the production of pharmaceuticals for the treatment of celiac disease. In addition, they can be employed to design and produce beneficial recombinant products based on the other papain-like cysteine proteases.

## Figures and Tables

**Table 1:** Expression of recombinant Triticain-α constructs.

Recombinant Protein	6HIS-Tag Position	AA ^a^	Strain	Expression Vector, Phenotype	Localization of the Product	Estimated Yield ^b^ (mg/L)	Specific Productivity ^c^ (µg/OD)
*Escherichia coli*							
6HIS-Triticain-α	*N*-terminal	469	BL21(DE3)	pET42-6HIS-Triticain-α	inclusion bodies	23.2 ± 1.88	11.1 ± 0.9
		469	Rosetta gami B (DE3)	pET15-6HIS-Triticain-α	inclusion bodies	17.2± 6.64	12.66 ± 4.34
pelB-Triticain-α-GM-6HIS	*C*-terminal	359	BL21(DE3)	pET26-Triticain-α-GM-6HIS	inclusion bodies	26.7 ± 2.76	18.4 ± 1.9
6HIS-Triticain-α-GM	*N*-terminal	345	BL21(DE3)	pET28-6HIS-Triticain-α-GM	inclusion bodies	61 ± 5.3	23 ± 2.0
		345	Rosetta gami B (DE3)	pET15-6HIS-Triticain-α-GM	inclusion bodies	21.4 ± 2.88	13.4 ± 1.8
					soluble fraction	17.3 ± 2.24	10.8 ± 1.4
		335	JM109	pQE-6HIS-Triticain-α-GM	inclusion bodies	34.2 ± 5.13	15.13 ± 2.27
Triticain-α-GM-6HIS	*C*-terminal	336	BL21(DE3)	pET28-Triticain-α-GM-6HIS	inclusion bodies	26.8 ± 0.91	11.8 ± 0.4
		334	Rosetta gami B (DE3)	pET15-Triticain-α-GM-6HIS	inclusion bodies	0	0
					soluble fraction	5.15 ± 0.5	3.55 ± 0.35
Triticain-α-GM	none	325	BL21(DE3)	pET26-Triticain-α-GM	inclusion bodies	32.8 ± 1.6	16.4 ± 0.8
		325	Rosetta gami B (DE3)	pET15-Triticain-α-GM	inclusion bodies	29.6 ± 1.1	12.05 ± 0.45
6HIS-Triticain-α-CatD	*N*-terminal	240	BL21(DE3)	pET28-6HIS-Triticain-α-CatD	inclusion bodies	174.6 ± 4.5	58.2 ± 1.5
		240	Rosetta gami B (DE3)	pET15-6HIS-Triticain-α-CatD	inclusion bodies	43.8 ± 0.26	25.85 ± 0.15
		231	JM109	pQE-6HIS-Triticain-α-CatD	inclusion bodies	72.2 ± 6.37	41.96 ± 3.54
Triticain-α-CatD-6HIS	C-terminal	228	BL21(DE3)	pET30-Triticain-α-CatD-6HIS	inclusion bodies	23.5 ± 5.36	11.05 ± 2.55
		227	Rosetta gami B (DE3)	pET15-Triticain-α-CatD-6HIS	no expression	–	–
Triticain-α-CatD	none	220	BL21(DE3)	pET26-Triticain-α-CatD	inclusion bodies	12.35 ± 5.14	5.8 ± 2.4
		220	Rosetta gami B (DE3)	pET15-Triticain-α-CatD	inclusion bodies	5.4 ± 0.79	2.45 ± 0.35
GST-Triticain-α-CatD	none	446	BL21	pGEX-GST-Triticain-α-CatD	inclusion bodies	150 ± 13.3	37.28 ± 3.32
6HIS-expP-Triticain-α-CatD	*N*-terminal	257	BL21(DE3)	pET15-6HIS-expP-Triticain-α-CatD	no expression	–	−
			Rosetta gami B (DE3)	pET15-6HIS-expP-Triticain-α-CatD	inclusion bodies	19.24 ± 0.54	8.87 ± 0.25
*Pichia pastoris*							
Triticain-α-GM	none	332	GS115	pPIC9-Triticain-α-GM, Mut+	growth medium	92 ± 12	−
			GS115	pPIC9-Triticain-α-GM, Muts	growth medium	164 ± 8	−
			GS115	pPIC9(pPIC9K)-Triticain-α-GM, Mut+	growth medium	178 ± 6	−
			GS115	pPIC9(pPIC9K)-Triticain-α-GM, Muts	growth medium	276 ± 28	−

^a^ Denotes amount of amino acid residues. ^b,c^ Determined as described in Materials and Methods Section.

**Table 2:** Co-expression of catalytic domain of Triticain-α with folding chaperones.

Recombinant Protein/Chaperone	6HIS-Tag Position in Catalytic Domain	Co-Expressing Vectors	Localization of the Product		
			Cell Growth Conditions		
			37 °C, 3 h	28 °C, 15 h	18 °C, 20 h
BL21(DE3)					
6HIS-Triticain-α-CatD/6HIS-Triticain-α ProD	N-terminal	pET28-6HIS-Triticain-α-CatD	inclusion bodies	−	−
		pQE80-6HIS-Triticain-α-ProD	inclusion bodies, partially soluble		
Triticain-α-CatD-6HIS/6HIS-Triticain-α ProD	C-terminal	pET30-Triticain-α-CatD-6HIS	inclusion bodies	−	−
		pQE80-6HIS-Triticain-α-ProD	inclusion bodies, partially soluble		
Triticain-α-CatD/6HIS-Triticain-α ProD	none	pET26-Triticain-α-CatD	inclusion bodies	−	−
		pQE80-6HIS-Triticain-α-ProD	inclusion bodies, partially soluble		
6HIS-Triticain-α-CatD C154A/6HIS-Triticain-α ProD	N-terminal	pET28-6HIS-Triticain-α-CatD C154A	inclusion bodies	inclusion bodies	−
		pQE80-6HIS-Triticain-α-ProD	inclusion bodies, partially soluble	inclusion bodies, partially soluble	
Triticain-α-CatD C154A-6HIS/6HIS-Triticain-α ProD	C-terminal	pET30-Triticain-α-CatD C154A-6HIS	inclusion bodies	inclusion bodies	−
		pQE80-6HIS-Triticain-α-proD	inclusion bodies, partially soluble	inclusion bodies, partially soluble	
Triticain-α-CatD C154A/6HIS-Triticain-α ProD	none	pET26-Triticain-α-CatDC154A	inclusion bodies	inclusion bodies	−
		pQE80-6HIS-Triticain-α-proD	inclusion bodies, partially soluble	inclusion bodies, partially soluble	
6HIS-Triticain-α-CatD C154A/6HIS-HSP70A1B	N-terminal	pET28-6HIS-Triticain-α-CatDC154A	−	inclusion bodies	−
		pQE80-6HIS-HSP70A1B		soluble, partially inclusion bodies	
Triticain-α-CatD C154A-6HIS/6HIS-HSP70A1B	C-terminal	pET30-Triticain-α-CatD C154A-6HIS	−	no expression	−
		pQE80-6HIS-HSP70A1B		soluble	
Rosetta gami B (DE3)					
6HIS-Triticain-α-CatD/6HIS-Triticain-α ProD	N-terminal	pET15[6HIS-Triticain-α-CatD+ 6HIS-Triticain-α-ProD]	−	−	inclusion bodies
					soluble

## Supplementary Materials

Supplementary materials can be found at www.mdpi.com/1422-0067/18/7/1395/s1.

## Abbreviations

Ac-PLVQ-AMC	acetyl-(PLVQ)-7-amino-4-methylcoumarin
CD	celiac disease also known as celiac sprue
extP	16-mer folding peptide (NYEEVIKKYRGEENF) from *Plasmodium falciparum* cysteine protease falcipain-2
GST	glutathione *S*-transferase
ORF	open reading frame
SDS-PAGE	sodium dodecyl sulphate polyacrylamide gel electrophoresis
PB	sodium phosphate buffer
pelB	N-terminal bacterial periplasmic signal sequence
Triticain-α-CatD	Triticain-α catalytic domain
Triticain-α-GM	Triticain-α lacking signal peptide and granulin domain
Triticain-α-ProD	Triticain-α prodomain
