# Supplementary material for: Rational Design of Recombinant Papain-Like Cysteine Protease: Optimal Domain Structure and Expression Conditions for Wheat-Derived Enzyme Triticain-α
Source: Int J Mol Sci. 2017 Jun 29;18(7):1395. doi: 10.3390/ijms18071395 (PMC5535888; doi:10.3390/ijms18071395)
Supplement: Supplementary file 1 [file ijms-18-01395-s001.pdf]

Supplementary Materials:

Rational Design of Recombinant Papain-Like Cysteine Protease: Optimal Domain Structure and Expression Conditions for Wheat-Derived Enzyme Triticain-α

Neonila V. Gorokhovets, Vladimir A. Makarov, Anastasiia I. Petushkova, Olga S. Prokopets, Mikhail A. Rubtsov, Lyudmila V. Savvateeva, Evgeni Yu. Zernii and Andrey A. Zamyatnin Jr.

Table S1. Oligonucleotides used for cloning of Triticain-α expression vectors.

Construct	Oligonucleotide (Forward/Reverse)*
pET26-Triticain-α-GM	5′-TATACATATGTCGATCGTGTCGTACGG/
	5′-TATTAAGCTTTTAGCCCGTCTTCGTCGGG
pET28-6HIS-Triticain-α-GM	5′-TATACATATGTCGATCGTGTCGTACGG/
	5′-TATTAAGCTTTTAGCCCGTCTTCGTCGGG
pET28-Triticain-α-GM-6HIS	5′-TATACCATGGCGGACATGTCGATCGTGTCG/
	5′-TATTCTCGAGGCCCGTCTTCGTCGGGTAG
pET26-pelB-Triticain-α-GM-6HIS	5′-TATACCATGGCGGACATGTCGATCGTGTCG/
	5′-TATTCTCGAGGCCCGTCTTCGTCGGGTAG
pQE80-6HIS-Triticain-α-CatD	5′-TGGATCCCTGCCGGAGACCGTCG/
	5′-TATTAAGCTTTTAGCCCGTCTTCGTCGGG
pET28-6HIS-Triticain-α-CatD	5′-TATCATATGCTGCCGGAGACCGTCG/
	5′-TATTAAGCTTTTAGCCCGTCTTCGTCGGG
pET26-Triticain-α-CatD	5′-TATCATATGCTGCCGGAGACCGTCG/
	5′-TATTAAGCTTTTAGCCCGTCTTCGTCGGG
pQE80-6HIS-Triticain-α-ProD	5′-ATGGATCCATCGTGTCGTACGGGGAG/
	5′-TATTAAGCTTTTACTCCTCGTTGTCGTCG
pET30-Triticain-α-CatD-6HIS	5′-TATCATATGCTGCCGGAGACCGTCG/
	5′-TTCTCGAGGCCCGTCTTCGTCGGGT
pGEX-GST-Triticain-α-CatD	5′-ATGGATCCTGCCGGAGACCGTCGAC/
	5′-TTCTCGAGTTAGCCCGTCTTCGTCGG
pET15-6HIS-Triticain-α-GM	5′-TATACATATGTCGATCGTGTCGTACGG/
	5′-TTCTCGAGTTAGCCCGTCTTCGTCGG
pET15-Triticain-α-CatD -6HIS	5′-ATACCATGGCGCTGCCGGAGACCGTCG/
	5′-ATTCTCGAGTCAGTGGTGGTGGTGGTGGTGGCCCGTCTTCGTCGGGT
pET15-Triticain-α-GM-6HIS	5′-TATACCATGGCGGACATGTCGATCGTGTCG/
	5′-ATTCTCGAGTCAGTGGTGGTGGTGGTGGTGGCCCGTCTTCGTCGGGT
pPIC9-Triticain-α-GM	5′-TGAATTCTCCATCGTGTCGTACGGG/
	5′-ATTGCGGCCGCTTAGCCCGTCTTCGTCGG
pPIC9K-Triticain-α-G	5′-TGAATTCTCCATCGTGTCGTACGGG/
	5′-ATTGCGGCCGCTTAGCCCGTCTTCGTCGG
	Mutagenic Megaprimer:
	5′-GCCGGAGACCGTCGATTGGAGGAAGAAGG
pET15-6HIS-extP-Triticain-α-CatD	5′-TTACATATGAATTATGAAGAAGTTATAAAAAAATATAGAGG/
	5′-ATTCATATGGAAATTTTCTTCTCCTCTATATTTTTTTATAA
pET15-6HIS-extP-Triticain-α-CatD^{C154A}	5′-TTACATATGAATTATGAAGAAGTTATAAAAAAATATAGAGG/
	5′-ATTCATATGGAAATTTTCTTCTCCTCTATATTTTTTTATAA
pET15[6HIS-Triticain-α-CatD+6HIS-Triticain-α-ProD]	5′-ATAAGATCTAAATCATAAAAAATTTATTTGC/
	5′-ATAAGATCTATTCTCACCAATAAAAAACG

* Restriction sites are underlined
